# Voices and Thoughts in Psychosis: An Introduction

**DOI:** 10.1007/s13164-015-0288-6

**Published:** 2015-10-21

**Authors:** Sam Wilkinson, Ben Alderson-Day

**Affiliations:** 1 Department of Philosophy, University of Durham, Durham, UK; 2 Department of Psychology, University of Durham, Durham, UK

## Abstract

In this introduction we present the orthodox account of auditory verbal hallucinations (AVHs), a number of worries for this account, and some potential responses open to its proponents. With some problems still remaining, we then introduce the problems presented by the phenomenon of thought insertion, in particular the question of how different it is supposed to be from AVHs. We then mention two ways in which theorists have adopted different approaches to voices and thoughts in psychosis, and then present the motivation and composition of this special issue.

## Introduction

Auditory verbal hallucinations (AVHs), namely, hearing voices in the absence of a speaker, are a common symptom of psychosis that affect approximately 75 % of people with schizophrenia (Bauer et al. 2011; Nayani and David 1996). AVHs are also seen in a range of other psychiatric disorders (including bipolar disorder, post-traumatic stress disorder and anorexia disorder) and are experienced by a minority of the non-clinical population (Beavan et al. 2011; Johns et al. 2014). Not only does the context (clinical or otherwise) in which AVHs occur vary, but the phenomenon’s properties, taken in isolation, vary enormously as well. Jones captures this heterogeneity nicely in the following.

The term AVH encapsulates a diverse phenomenological experience, which may involve single and/or multiple voices, who may be known and/or unknown, speaking sequentially and/or simultaneously, in the first, second, and/or third person and which may give commands, comments, insults, or encouragement. (2010, p.566).

AVHs can be deeply troubling for those who experience them, and a greater understanding of them as a phenomenon finds clear motivation in the desire to improve management and treatment of an often very distressing experience. But beyond this clinical motivation, there exist a number of theoretical ones, too. There is nothing *obvious* about hearing voices, which is to say: nothing that we know about human beings clearly predicts the presence, prevalence and variety of AVHs. Thus we can turn this on its head and ask: what does the presence, prevalence and variety of AVHs tell us about human beings in general, and human cognition in particular? What would human beings have to be like in order to give rise to the phenomena that we see when people report AVHs? Some theorists, as we are about to see, make very strong claims about the nature of the human mind and brain, in trying to account for the presence of AVHs.

In this introduction we set the scene by presenting the orthodox account of AVHs, which makes use, not only of the notion of self-monitoring, but also of the notion of inner speech. We then present a number of worries for this account, and some potential responses open to its proponents. With some problems still remaining, we then introduce the problems presented by the phenomenon of thought insertion, not least the question of how different it is supposed to be, as a phenomenon, from AVHs. We then mention two ways in which theorists have adopted different approaches to voices and thoughts in psychosis, and present the motivation and composition of this special issue.

## The Orthodox Account of AVHs and its Detractors

### Self-Monitoring Accounts of AVH

By far the most popular approach in recent decades has been to account for AVHs in terms of a problem with self-monitoring (Frith 1992, Fritht et al. 2000, Seal et al. 2004). The starting point (as with all of these accounts, whether implicitly or explicitly) is a claim about the human nervous system. In this case, the claim is that your nervous system is constantly in the business of trying to work out what sensory stimulation has been produced by itself, or by the outside world. This is called self-monitoring.

How is self-monitoring actually achieved? The first theorist to postulate a self-monitoring mechanism was arguably von Hemlholtz (1866). His concern, however, was with the following problem. When an image moves across the retina, how does our nervous system know whether it is the world moving across our eyes or our eyes moving across the world? Helmholtz suggested that our nervous system can tell the difference because when our eyes move there is a motor command. More specifically, information about the motor command is used by the nervous system to predict the sensory consequences that would be produced by the eye movement. If the predicted and actual sensory consequences match then the nervous system infers that the change was self-generated and the conscious percept is adjusted accordingly. We can see exactly what happens when there is no such motor command, and hence no such adjustment, when we press on our eye with our finger. When we do this, the world itself seems to tilt and shake. This is because the nervous system is, at some level, taking the world to be moving across the eye, rather than the eye across the world.

The same basic idea underlies the self-monitoring mechanism postulated to account for the symptoms of schizophrenia, including AVHs. The easiest symptoms for which to introduce the account are delusions of control, since it is clear that, if anything involves motor commands, and indeed sensory consequences, then bodily actions do. In delusions of control, a subject may perform actions that are in keeping with her plans and intentions (for example, she might brush her hair), but she claims that somebody else is controlling her. Frith and Done (1989) took this to be the result of a mismatch between the predicted and actual sensory consequences of the bodily movement and so the movement is attributed to an external source.

Whereas in Helmholtz’s example, the recognition by the nervous system that a certain stimulus is self-produced causes a correction of the conscious percept, in more typical bodily motor control, where sensory consequences are proprioceptive and tactile, it results in sensory attenuation. The benefit of this attenuation is clear: your nervous system needs to pay attention to stimuli that come from the (potentially threatening) outside world, not the self-produced stimuli that will tend to be harmless and irrelevant.

Various data suggest that something goes wrong with this monitoring and subsequent attenuation in people with a diagnosis of schizophrenia (Blakemore et al. 2000; Ford and Mathalon 2004). Perhaps the most striking such datum is the apparent finding that people with schizophrenia can tickle themselves. Typical subjects, in contrast, cannot tickle themselves because their nervous systems accurately monitor, and successfully attenuate, the sensory consequences of the tickling movements (Blakemore et al. 2000).

How is this applied to AVHs? The basic idea is that in schizophrenia or, more generally, psychosis, there is one deficit concerning the monitoring of self-produced stimuli, and that different symptoms arise depending on what kinds of stimuli are failing to be properly self-monitored. In delusions of control it is physical action that fails to be properly monitored, whereas in AVH it is so-called “inner speech”. Although different theorists have different views about what inner speech is (see Alderson-Day and Fernyhough 2015 for a comprehensive review) it is, roughly, the production and experience of speech without any overt articulation. It is, so to speak, “that little voice inside one’s head”, often associated with verbal thinking.

Thus we get the orthodox view of AVHs: they are to be understood as the result of disrupted monitoring of inner speech.

### Problems for Self-Monitoring Accounts

One could criticise self-monitoring accounts in at least two different ways. One way involves saying that, regardless of whether the mechanism that is postulated could potentially account for what we want to account for, the mechanism postulated somehow does not make sense. The other involves admitting that the mechanism (in this case badly monitored inner speech) makes sense, in principle, but does not account for what we need it to account for.

To take these in turn, a prima facie objection would be to deny that inner speech is any kind of action or motor process. It therefore cannot be self-monitored, given that self-monitoring exploits information from motor commands (often referred to as “efference copies”) to inform its predictions. As a result, the idea that inner speech can be misattributed, just like any other action, does not get off the ground.

However, arguably, there are reasonable grounds to think of inner speech as an action, with its roots in the motor system (see Jones and Fernyhough 2007). Although it is not intuitively obvious that inner speech is motoric, this has been empirically supported by several electromyographical (EMG) studies (e.g. Jacobsen 1931) which picked up muscular activity during inner speech. Later experiments have also made the connection between inner speech and AVH, showing that similar muscular activation is involved in healthy inner speech and AVH (Gould 1948). The involvement of motoric elements in both inner speech and in AVH is further supported by findings from Gould (1950), who showed that when his subjects reported hearing voices, subvocalizations occurred which could be picked up with a throat microphone. This is unlikely to be the case for all AVH (Jones 2010), but suggests that motor involvement in both inner speech and AVH should not be ruled out.

Another more pressing criticism grants that inner speech is (or might be) motoric, but questions whether it would ever be the kind of thing to be monitored. Since it is not an overt action occurring in three-dimensional space, it is not clear that it generates any sensory consequences, at least not in anything like the usual sense. Nor is it clear that there can be a mismatch between the actual and predicted sensory consequences; a mismatch that is central to the classic self-monitoring account. There are various ways of amending the inner speech model to avoid such a problem: for example, one might argue that inner speech is simply an attenuated form of external speech, using the same (or similar) neural architecture and thus generating its own predictive models. Alternatively, certain psycholinguistic approaches associate the experience of inner speech with the sensory prediction itself (e.g. Scott 2013), thus avoiding the need to posit a separate sensory prediction for an internal state that is “compared” to inner speech. Such approaches still need to explain how misattribution can occur, but offer a way of retaining the speech-motor basis of AVH. Nevertheless, there are arguably no convincing and comprehensive accounts of where inner speech sits within a self-monitoring model (Alderson-Day and Fernyhough 2015), and its proponents have in recent years moved away from posting it as an explanation for AVH (e.g. Frith 2012).

The second family of criticisms grants (even if only for the sake of argument) that inner speech could, in principle, be self-monitored, and that this, in principle, could go awry, but denies that this explains AVHs. Following Wilkinson (2014), we call these criticisms or challenges the *varieties of AVH challenge* and *the auditory phenomenology challenge*.

The varieties of AVH challenge is, quite simply, that AVHs vary so much (as we saw at the beginning) that it seems unlikely that all of them are to be accounted for in terms of misattributed inner speech based on self-monitoring problems. One response to this is to say that the orthodox inner speech model accounts for at least some of the things we call AVHs (McCarthy-Jones et al. 2014a).

The auditory phenomenology challenge can be stated as follows. Granting, for the sake of argument, that AVHs are misattributed episodes of inner speech resulting from self-monitoring problems, we must explain how we get a transformation from.

the experience of the subject’s own inner voice […] often lacking acoustical properties such as pitch, timbre, and intensity into the experience of someone else’s voice with acoustical properties. (Cho and Wu 2013, p.2).

In short, how does inner speech become auditory?

There are various responses available to counter this worry. One is that a close inspection of the phenomenology of AVH suggests that perhaps not all AVHs are clearly auditory, and so, in order to account for these, we would not need to account for auditory phenomenology. (More on this later) Another response is to attack the premise of the argument, and deny that inner speech truly lacks all of these features in all cases. For example, one might insist that inner speech can and does have auditory phenomenology, and/or second-person pronoun use, and/or the representation of other agents (see, for example, Fernyhough 2004; Alderson-Day and Fernyhough 2015).

Nevertheless, a puzzle remains for theorists who posit the link between inner speech and AVH – if inner speech is misattributed, why does that result in an alien *voice*, and not an alien *thought*?

### Enter Thought Insertion

This brings us to another way in which the picture is complicated by (what is typically taken to be) a separate phenomenon, also commonly associated with diagnoses of schizophrenia, namely, the phenomenon of *thought insertion*. Thought insertion (TI) is often described as the phenomenon of experiencing someone else’s thoughts. One common approach has been to explain TI *also* in terms of misattributed inner speech; that is, the normal processes of self-monitoring fail in some way, and inner speech is not recognised as one’s own. But then, if inserted thoughts and AVHs are to be explained in the same way, then why are not they reported as being the same phenomenon?

One possibility is that they are actually the same underlying experience, but because the experience is highly unusual and hard to describe, it is being reported in different ways by different people. In support of this are examples of phenomena that appear to blur the lines between the two experiences: for instance, Bleuler (1950) also described examples of “soundless voices” and “audible thoughts” in the case reports of people with schizophrenia that he saw. Thus, it could be that experiencing inserted thoughts, and hearing soundless voices, are actually one and the same phenomenon; the same experience, interpreted and reported differently. This somewhat deflationary view would still need to explain individual differences in how the experiences are described, but at least offers a means to account for AVH and TI under one model.

Another possibility is that AVH and inserted thoughts represent variations of the same underlying experience, and that these variations (in, for example, perceptual phenomenology), drive different descriptions of AVH and TI. In support of this idea is the fact that AVH and TI often co-occur in people who report them (Nayani and David 1996), suggesting that people are choosing to describe phenomenologically separable experiences in separate terms. On this reading, silent voices and inserted thoughts are both the same phenomenon (at a suitable level of abstraction), but the explanatory challenge is to shed light on why they are reported as different phenomena, perhaps due to the context in which the phenomenon arises, or the nature of the phenomenon itself (whether this be a difference in the kind of self-monitoring failure, or the kind of inner speech).

Finally, AVH and TI could simply fall under different categories and require different explanations. While the two experiences often co-occur, they have different prevalence rates, with the former thought to be much more common (see Badcock 2015). Thought insertion, for example, is often classified in psychiatric terms as a kind of delusion; a matter of belief, rather than perception (as in the case of AVH). If they are separate phenomena, the self-monitoring theorist needs to explain why misattributions in inner speech apply to AVH and not TI (or vice versa).

### Updated Accounts of AVH

As the above concerns show, the self-monitoring orthodoxy is not entirely unproblematic. At best, it needs to be supplemented; at worst, it needs to be abandoned altogether. Contemporary research in voice hearing is starting to depart from this orthodoxy in exciting ways. Although the pressure is coming from two very different sources, both point towards something similar: namely, a need to account for the complexities, and varieties, of the phenomena in question. In particular, we need to be wary of the tendency to assume that all of the phenomena we call AVHs are one and the same phenomenon, explainable in terms of an overarching model.

One source of change comes from theoretical neuroscience. A new framework (see Clark 2013 for review) is viewing what the brain does in terms of knowledge-driven prediction. This framework either subsumes (Fletcher and Frith 2009) or dispenses with (Van Doorn et al. 2013; Adams et al. 2013) self-monitoring. If hallucinations and other experiences in psychosis are taken to involve errors in prediction, this has scope for explaining much more and to do so in a more fine-grained way, incorporating cutting-edge neurobiological (Corlett et al. 2010) and computational work (Friston 2010), and accommodating person-specific influences, such as life context and personal history. Within this framework, predictive processing can be disrupted in a number of ways, as a result of a number of causes, with the potential to generate several variations and subtypes of AVH and TI (Wilkinson 2014).

The other source of change comes from a renewed recognition of the importance of first-person experience in understanding AVH (McCarthy-Jones et al. 2014b; Sass 1994). Theorists in psychology and philosophy have been interacting more with clinicians, patients and even activists to develop a closer understanding of what it is like to hear a voice (e.g. Longden et al. 2012; Woods et al. 2015) putting direct pressure on the need to recognise complexity and heterogeneity in our understanding of psychosis. Not only are there the commonly differentiated symptoms, but, within AVHs alone there is an overwhelming degree of diversity. This has led many theorists to accept that, if models that understand AVH in terms of misattributed inner speech are tenable, they may only apply to a subset of experiences (Jones 2010). Along with increasing recognition of voice hearing in the non-clinical population (Larøi 2012), this has led to a broader and richer view of hallucinations and other abnormal experiences, and a renewed focus on revising standard models of AVH and TI.

## Motivation and Content of the Special Issue

It is against this backdrop that this special issue finds its motivating questions. In this section we present these, and show of how various contributors address them.

First off: If we experience thoughts in our head, how can they seem to not be our own? That is the central paradox of thought insertion. To understand this, though, we need to explore what thoughts are and how we know them in the first place. In his paper, **Johannes Roessler** outlines two views about knowledge of our own thoughts, attributed to Gilbert Ryle. The first, is that we are “alive” to our own thoughts in the “serial process” of thinking, and the second is that we can “eavesdrop” on our inner speech, and interpret our own utterances in much the same was as we interpret the utterances of others. Roessler argues that the former is the correct account of how we know that (and what) we are thinking for the vast majority of the time. On this reading, unusual cases such as thought insertion depend on a breakdown in this experience, such that thoughts are not intelligible in terms of an ongoing serial process.

One consequence of this view is that it reframes the standard way of thinking about thought insertion (Gallagher 2000, 2004). The standard approach wants to explain thought insertion in terms of certain features of the phenomenology of a thought. According to the standard account, there are two ways in which a thought can be experienced as “mine”. One is that it can be experienced as falling within my psychological boundaries; the other, is that I can experience myself as somehow its agent, or source. The first sense is referred to as “ownership”, and the second as “agency” (Gallagher 2000) or more recently, as “authorship” (Bortolotti and Broome 2009). Due to disruptions in the phenomenology of thought, perhaps attributable to self-monitoring deficits, patients who report an inserted thought experience that thought as retaining “ownership” (so “mine” in one sense), but lacking “agency” (so “not mine” in another).

If Roessler and Ryle are right, then this approach is too decontextualized (although it may be along the right lines). A thought is not a free-standing experience. It is not, therefore, describing or explaining some unusual aspect to the phenomenology of a thought, *qua* an experience, that will provide the account of thought insertion. Rather, thinking is a deeply contextually-embedded, serial process that one is usually “alive to” when one is doing it. Thought insertion would then result from a disruption to this practical awareness of what one is doing. Roessler does not go as far as to suggest a cause of such disruption, but one could hypothesise that various different things – and different *kinds* of thing - could act as contributors, from overwhelmingly strong emotions to deficits in working memory. It is worth noting that standard accounts that appeal to retained ownership but lost agency are often criticised for failing to account for why some thoughts that, intuitively, lack agency, are not reported as inserted. Among such thoughts are obsessive or intrusive thoughts. In contrast, on a view that built on Roessler’s claims, one would be “alive to” intrusive and obsessive thoughts even if one did not feel that one had brought them about agentively or endorsed their content.

In her paper, **Rachel Gunn** also questions orthodox accounts of thought insertion. She proposes that accounts of thought insertion that posit ownership without agency miss the point about the experience, and, in particular, are not going into enough detail about the different things that “ownership” can mean. Drawing on a range of vivid, first-person accounts, Gunn argues that inserted thoughts lack what she calls “personal ownership”, that is, a sense of ownership where thoughts are consistent with one’s own unique experience and context, a sense of “myness” that accompanies our thinking the rest of the time. Although methodologically Roessler and Gunn’s contributions are worlds apart, their take-home messages for our understanding of thought insertion have strong continuities.

Whereas Gunn focuses on thought insertion as an experience (granted, one that is deeply context-dependent), **Pablo Lopez-Silva**, in contrast, focuses on thought insertion as a kind of *delusion*. That is to say, the question of explanatory relevance is not: “What is this person experiencing?”, but rather: “What might lead someone, not so much to experience their thoughts as inserted, but to form the *belief* that a thought has been inserted?” This belief need not be a straightforward endorsement of strange phenomenology accompanying an episode of thinking (where *what* is thought, the thought content, is not explanatorily relevant), but rather a rational appraisal to a particular thought content. Thus, for Lopez-Silva, the content of the inserted thought is of crucial explanatory relevance. TI is not simply the product of a low-level disruption to phenomenology, according to which any old thought, in principle, could be experienced as inserted. More specifically, the core claim is that the delusion of thought insertion is one which protects the individual from ego-dystonic cognitions – thoughts that clash with our own sense of “me”. Furthermore, Lopez-Silva argues that we must pay more attention to the role that affective disturbances play in driving and generating ego-dystonic thought contents.

Such disturbances play a key role in the tradition of phenomenological psychopathology, within which the article by **Peter Handest and colleagues** firmly falls**.** In their article, they offer an introduction to the work of Klosterkotter, Conrad, Sass, and Parnas on how a range of disruptions to thoughts and perceptions can develop into auditory hallucinations in the context of schizophrenia. Drawing on the work of Conrad in particular, they posit that gradual changes in the salience of the world around an individual (*apophany*), the anxiety that the world holds (*trema*), and the tendency to reflexively focus on one’s own experience (*hyper-reflexivity*) combine to give rise to a situation where thoughts are given their own external agency and perceptual quality. In such a context, both hearing voices and having inserted thoughts are part of the same overall disruption to the self (*ipseity*) that occurs in psychosis, which Handest et al. illustrate with a number of case examples.

Two papers that also seek to bridge the gap between AVH and TI are provided by Humpston & Broome and Badcock, one focusing on phenomenology; the other, on neuropsychology. In the first, **Clara Humpston & Matthew Broome** use first-person accounts to emphasise the phenomenological continuity between experiences of voices and thoughts in psychosis. While recognizing the place of appraisal and elaboration, they reject the idea that TI should primarily be considered a delusion (contra, e.g. Lopez-Silva 2015) and place it on a quasi-perceptual continuum with soundless voices and auditory hallucinations. For Humpston & Broome, though these experiences differ in audibility, they share a number of characteristics in terms of their disrupted agency and ownership, and they suggest that, in practice, the majority of patients will have a mixed experience of both of these phenomena.

**Johanna Badcock**, meanwhile, offers a glimpse of how combined accounts of AVH and TI might be accommodated in a neuropsychological model. Neuroimaging evidence from ordinary voice perception suggests that multiple processing streams in the brain are responsible for recognising voice identity, location and other features. Badcock uses these findings, along with epidemiological data, to propose a parallel processing framework that can account for alien experiences of voices and thoughts. Specifically, she argues that both AVH and TI result from disruptions to neural networks responsible for audition and language, but that their phenomenology will vary depending on which specific network components are affected. She also places this model within the predictive processing approach (Clark 2013; Wilkinson 2014), suggesting that it can be integrated with more global models of perception and action.

Finally, there are questions that are not related to thought and thought disturbance, but more to orthodox accounts of AVH. In particular, if voices result (in some way, at least) from inner speech, how do we account for the “otherness” of their identity? And what is being compared when we generate an episode of inner speech? Drawing on arguments from Gallagher (2004) and Wu (2012), **Gregory** argues that we can make more sense of AVH as misattributed *imagined* speech (or auditory verbal imagery), rather than inner speech. In turn, inner speech has more in common with actual speech than with verbal imagery. This approach touches on an older tradition of thinking about AVH, and a set of unresolved questions regarding the relationship between inner speech and imagery. Regarding the former, imagery-based explanations of AVH in fact predate inner speech models (Mintz and Alpert 1972), although evidence of imagery processes differing between people with and without AVH has been somewhat inconsistent (Aleman et al. 2003). Regarding the latter, inner speech would be considered by many as distinct from imaginative processes. However, the lines we draw between vivid instances of inner speech and verbal content in auditory imagery are not at all clear, especially if we allow for features such as dialogue and tone in our concept of inner speech (Alderson-Day and Fernyhough 2015).

In stark contrast, **Peter Langland-Hassan** offers a solid defence of a revised inner speech model, via an emphasis on sensory attenuation rather than sensorimotor comparison. Key to self-monitoring accounts is the idea that sensory cortex is typically dampened in response to self-generated stimuli. Langland-Hassan draws on evidence of this to suggest that we can account for AVH without reference to the comparison between prediction and action traditionally included in self-monitoring accounts. He also extends this account to incorporate thought insertion and thought disorder, with reference to semantic errors in Wernicke’s aphasia. In this condition, the content of what a person says often differs considerably from their intended meaning, as a function of the aphasia. Langland-Hassan uses this example of *semantic* self-monitoring failure to highlight how the interactions between speech production and perception lie not just in prediction of sensory consequences, but also in the expression of content: the detection of what is being said, just as much as what it sounds like. This opens up a new way of thinking about unusual voices and thoughts that depart from the “train” of thought, whether that is considered a serial operation (Roessler), a violation of personal ownership (Gunn), or an example of ego-dystonia (Lopez-Silva).

## Directions for Future Research

The phenomena of AVH and thought insertion pose unusual explanatory challenges, in that they force us to examine fundamental questions about thought, perception, and agency. The articles collected here demonstrate the breadth of inquiry necessary for examining what it is to experience an AVH or have an inserted thought. Moreover, many of these contributions highlight the importance once again of closely examining first-person descriptions of unusual experiences, and making sure that convenient taxonomies do not hide a continuum of similarities across superficially separate phenomena, or indeed lump together distinct but superficially similar phenomena. Needless to say, further work from philosophers, psychologists, psychiatrists, etc. is essential to answering the questions posed by voices and thoughts in psychosis.

Some of these questions are highly general, focusing more on human cognition generally, and only having an indirect impact on our understanding of psychosis. For example, we might ask: is the nervous system really just a prediction machine in the way that some recent theorists suggest? If so, then self-monitoring theories are along the right lines, but the basic principle needs to apply beyond prediction of *self-produced* stimuli to prediction of *all* stimuli. This clearly has potential impact for our understanding of psychosis, since if prediction does not rely on motor commands, and psychosis is a problem with this sort of low-level prediction, then explanations of psychotic symptoms need not be restricted to phenomena that involve motor commands (Wilkinson 2015). Another question we might ask is: What is the relationship between inner speech and thought? There does seem to be an interesting relationship here, but not all inner speech is thinking (see Roessler 2015), and not all thinking is in inner speech. In which case, it’s a short step from this to the question: might some cases of thought insertion be non-verbal (e.g. imagistic)? If the answer to this is yes, then inner speech cannot plausibly be implicated in (at least those cases of) thought insertion.

Other questions are more directly tied to psychosis. For example, we might ask: What is the relationship between trauma and psychotic symptoms like AVH and voice hearing? If trauma is a strong causal factor, by what mechanism does it exert the causal influence that it does? These questions are more typically the realm of clinical psychology and psychiatry journals, but it seems clear that important ground is made when philosophers, psychologists, psychiatrists and neuroscientists interact.
